# The impact of different alignment strategies on bone cuts in total knee arthroplasty for varus knee phenotypes

**DOI:** 10.1007/s00167-023-07351-w

**Published:** 2023-02-22

**Authors:** Benjamin L. Schelker, Céline S. Moret, Manuel P. Sava, Rüdiger von Eisenhart-Rothe, Heiko Graichen, Markus P. Arnold, Vincent Leclercq, Michael T. Hirschmann

**Affiliations:** 1 Department of Orthopedic Surgery and Traumatology, Kantonsspital Baselland, 4101 Bruderholz, Switzerland; 2 Department of Clinical Research, Research Group Michael T. Hirschmann, Regenerative Medicine and Biomechanics, University of Basel, 4001 Basel, Switzerland; 3 Department of Orthopedics and Sports Orthopedics, Klinikum Rechts der Isar, Technical University Munich, Ismaningerstr. 22, 81675 Munich, Germany; 4 Department of Arthroplasty, Sports Medicine and Traumatology, Orthopaedic Hospital Lindenlohe, 92421 Schwandorf, Germany; 5 LEONARDO, Hirslanden Klinik Birshof, Münchenstein, Switzerland; 6 Symbios, Yverdon-Les-Bains, Switzerland

**Keywords:** Knee, Arthroplasty, TKA, Alignment, Kinematic, Mechanical, Phenotype, Restricted, Anatomical, Bone cuts, Varus

## Abstract

**Purpose:**

The purpose of this study was to visualise the influence of alignment strategy on bone resection in varus knee phenotypes. The hypothesis was that different amounts of bone resection would be required depending on the alignment strategy chosen. Through visualisation of the corresponding bone sections, it was hypothesised, it would be possible to assess which of the different alignment strategies would require the least amount of change to the soft tissues for the chosen phenotype, whilst still ensuring acceptable alignment of the components, and thus could be considered the most ideal alignment strategy.

**Methods:**

Simulations of the different alignment strategies (mechanical, anatomical, constrained kinematic and unconstrained kinematic) in relation to their bone resections were performed on five common exemplary varus knee phenotypes. VAR_HKA_174° VAR_FMA_87° VAR_TMA_84°, VAR_HKA_174° VAR_FMA_90° NEU_TMA_87°, VAR_HKA_174° NEU_FMA_93° VAR_TMA_84°*,* VAR_HKA_177° NEU_FMA_93° NEU_TMA_87° and VAR_HKA_177° VAL_FMA_96° VAR_TMA_81°. The phenotype system used categorises knees based on overall limb alignment (i.e. hip knee angle) but also takes into account joint line obliquity (i.e. TKA and FMA) and has been applied in the global orthopaedic community since its introduction in 2019. The simulations are based on long-leg radiographs under load. It is assumed that a change of 1° in the alignment of the joint line corresponds to a displacement of the distal condyle by 1 mm.

**Results:**

In the most common phenotype VAR_HKA_174° NEU_FMA_93° VAR_TMA_84°, a mechanical alignment would result in an asymmetric elevation of the tibial medial joint line by 6 mm and a lateral distalisation of the femoral condyle by 3 mm, an anatomical alignment only by 0 and 3 mm, a restricted by 3 and 3 mm, respectively, whilst a kinematic alignment would result in no change in joint line obliquity. In the similarly common phenotype 2 VAR_HKA_174° VAR_FMA_90° NEU_TMA_87° with the same HKA, the changes are considerably less with only 3 mm asymmetric height change on one joint side, respectively, and no change in restricted or kinematic alignment.

**Conclusion:**

This study shows that significantly different amounts of bone resection are required depending on the varus phenotype and the alignment strategy chosen. Based on the simulations performed, it can, therefore, be assumed that an individual decision for the respective phenotype is more important than the dogmatically correct alignment strategy. By including such simulations, the modern orthopaedic surgeon can now avoid biomechanically inferior alignments and still obtain the most natural possible knee alignment for the patient.

## Introduction

Varus deformity of the knee is the most common angular deformity in the coronal plane. Today, there is an ongoing controversy about the optimal alignment strategy in total knee arthroplasty (TKA) to address this deformity [18, 23]. In TKA mechanical alignment (MA) aiming to restore neutral limb alignment by cutting the femur and tibia perpendicular to the ground results in an equal medial and lateral load distribution and therefore has been the target of choice [24]. Although MA has led to excellent implant survival rates, a significant proportion of patients remained dissatisfied with the functional outcome despite the use of advanced implant designs and improved precision of surgical technique [16]. Amongst others, one possible reason for dissatisfaction could be that a neutral alignment of the leg is not a natural alignment for all patients [24]. Solely 35.4% of the non-osteoarthritic population has a HKA of 180° ± 1.5° [7]. Similarly, Bellemans et al. [2] have shown that about 32% of male and 17% of female in the healthy population have a constitutional varus limb alignment (HKA < 177°). In these varus patients, the greater changes necessary with MA in terms of bony resections and ligament releases are thought to be a contributing cause of postoperative dissatisfaction after TKA. Moreover, applying the functional knee phenotypes identified by Hirschmann et al., it was shown that only 5, 20 and 51% of the normal population had a knee morphology and leg alignment analogous to the MA, anatomical (AA) and restricted kinematic alignment (rKA), respectively [9]. Hence, modern alignment strategies as the unrestricted kinematic alignment (KA) aim to achieve more natural kinematics and improve functional outcomes by restoring the native pre-arthritic alignment and preserving ligamentous structures [18]. However, there are yet conflicting results as to whether patients with a constitutional varus knee have better clinical results when the knee is left in varus [15, 23, 28]. It is still unknown to what degree the varus alignment should be maintained and what effects the different alignment strategies have on the knee and gait biomechanics. However, positioning the TKA in varus alignment could lead to faster implant failure, as unintentional varus alignment of components in the past has led to increased rates of aseptic loosening, early polyethylene failure and therefore revision surgery [14, 31].

Hence, the aim of this study was to perform a simulation study to illustrate (1) how the coronal limb alignment of the most common exemplary varus knee phenotypes is changed by current systematic and personalised alignment strategies and (2) whether these visualisations could be used to establish basic recommendations for selecting the best alignment strategy for those specific varus phenotypes. It was hypothesised that a patient with a preoperative varus limb alignment would undergo different bone resections depending on the specific knee phenotype and alignment strategy chosen. As the overall limb alignment would be altered differently in varus phenotypes depending on the alignment strategy used, the choice of alignment strategy would likely have a decisive influence on the resulting dynamic and loaded alignment of the knee. In contrast to previous research, this study was not designed to find the most appropriate alignment strategy for the majority of knees, but to illustrate the effects of the different alignment strategies on the individual phenotypes.

## Materials and methods

**Fig. 1:**
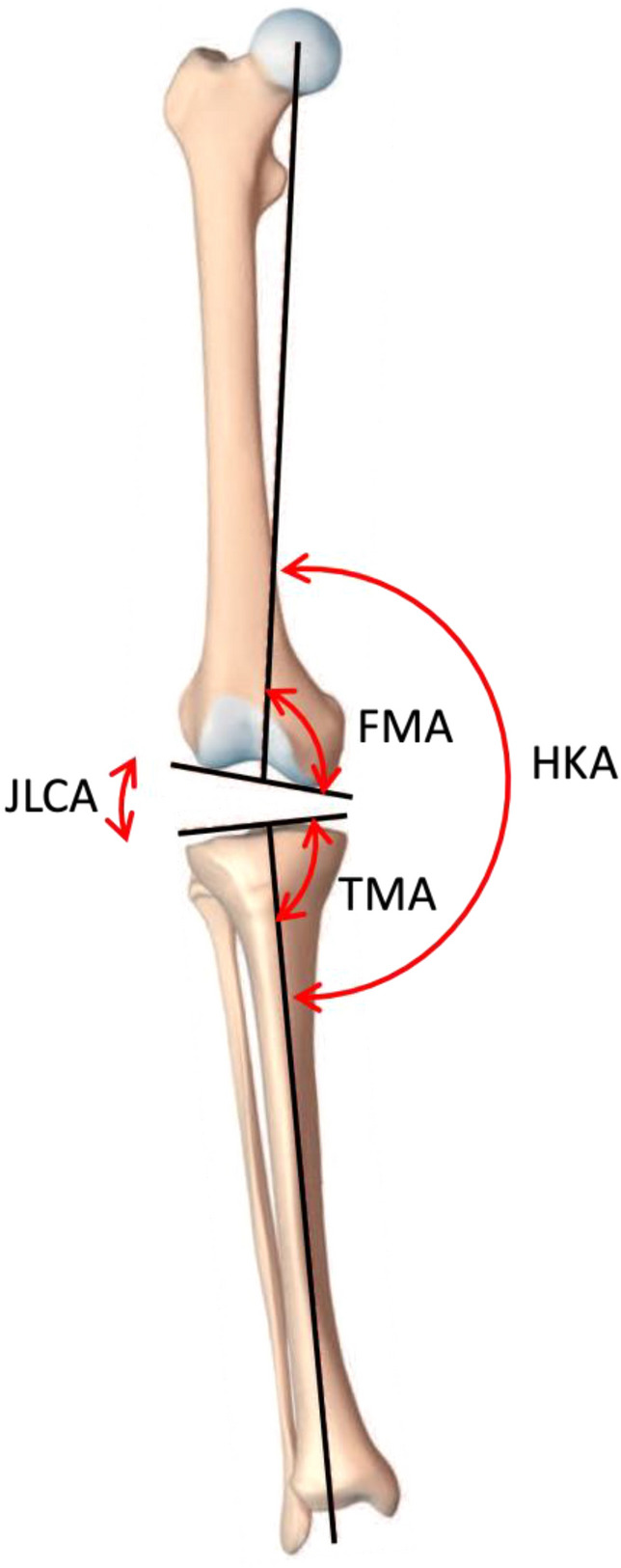
The hip–knee–ankle angle (HKA) is formed by the lines connecting the centre points of the femoral head, the knee and the talus; FMA is the angle between the mechanical axis of the femur and a tangent to the distal femoral condyles; TMA is defined as the angle between the mechanical axis of the tibia and a tangent to the proximal articular surface of the tibia. The joint line convergence angle (JLCA) is the angle between a tangent to the proximal articular surface of the tibia and the tangent of the femoral condyles

The coronal alignment of exemplary functional knee phenotypes is described and the imbalance of distal bone cuts and the resulting distal femoral joint line changes are assessed in this simulation study. The hip–knee–ankle angle (HKA), the mechanical femur angle (FMA) and the mechanical tibia angle (TMA) are displayed in Fig. 1. All angles are measured medially. Neutral (NEU) femoral and tibial as well as limb alignments are defined as 93° (± 1.5°) for the FMA, 87° (± 1.5°) for the TMA and 180° (± 1.5°) for HKA. Consequently, a value above 94.5° for FMA and 88.5° for TMA or above 181.5° for HKA corresponds to a valgus (VAL) alignment and a value below 91.5° for FMA and 85.5° for TMA, or below 178.5° for HKA corresponds to a varus (VAR) alignment. For better illustration, neutral angles are shown in green, varus in blue and valgus in red. A change in 1° in the joint line orientation is considered to correspond to 1 mm of distal condyle offset.

### Functional knee phenotypes

**Fig. 2:**
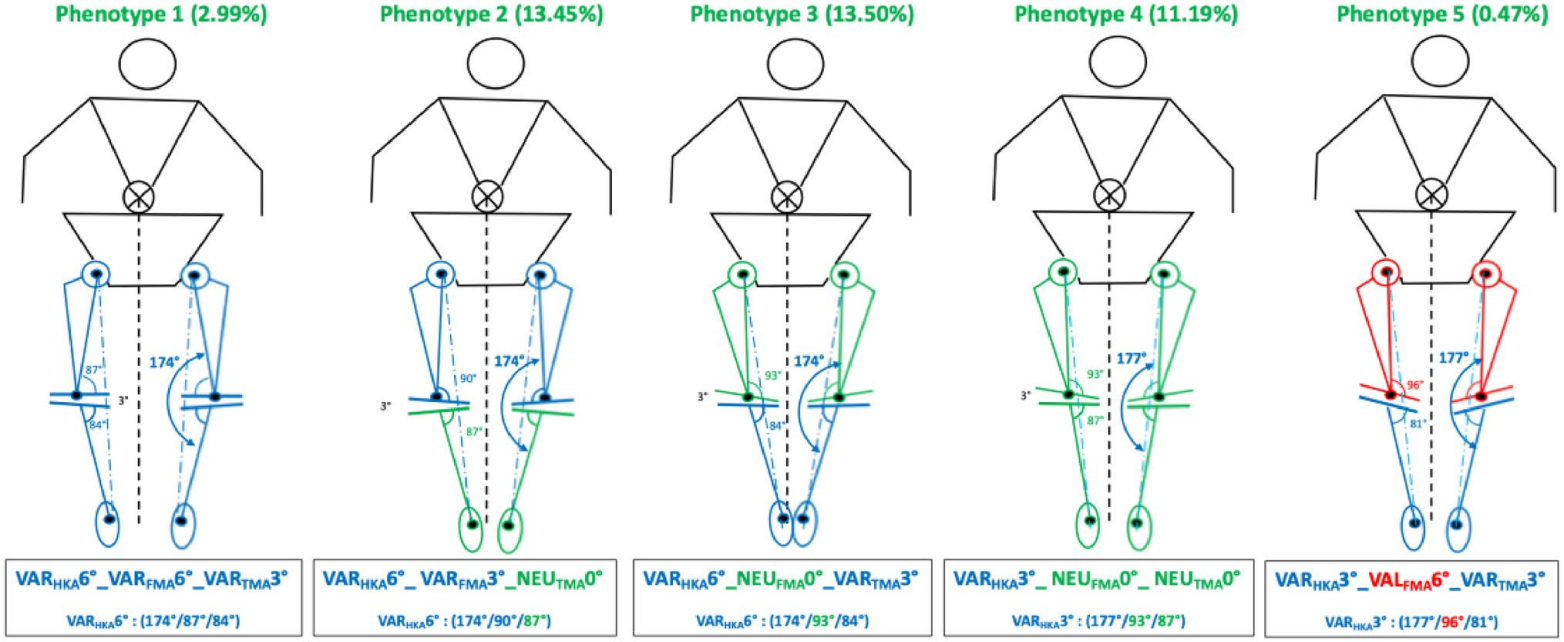
The five exemplary VAR phenotypes (STATIC). The indicated prevalences refer to the proportion of phenotypes in the population with varus-aligned knees

**Fig. 3:**
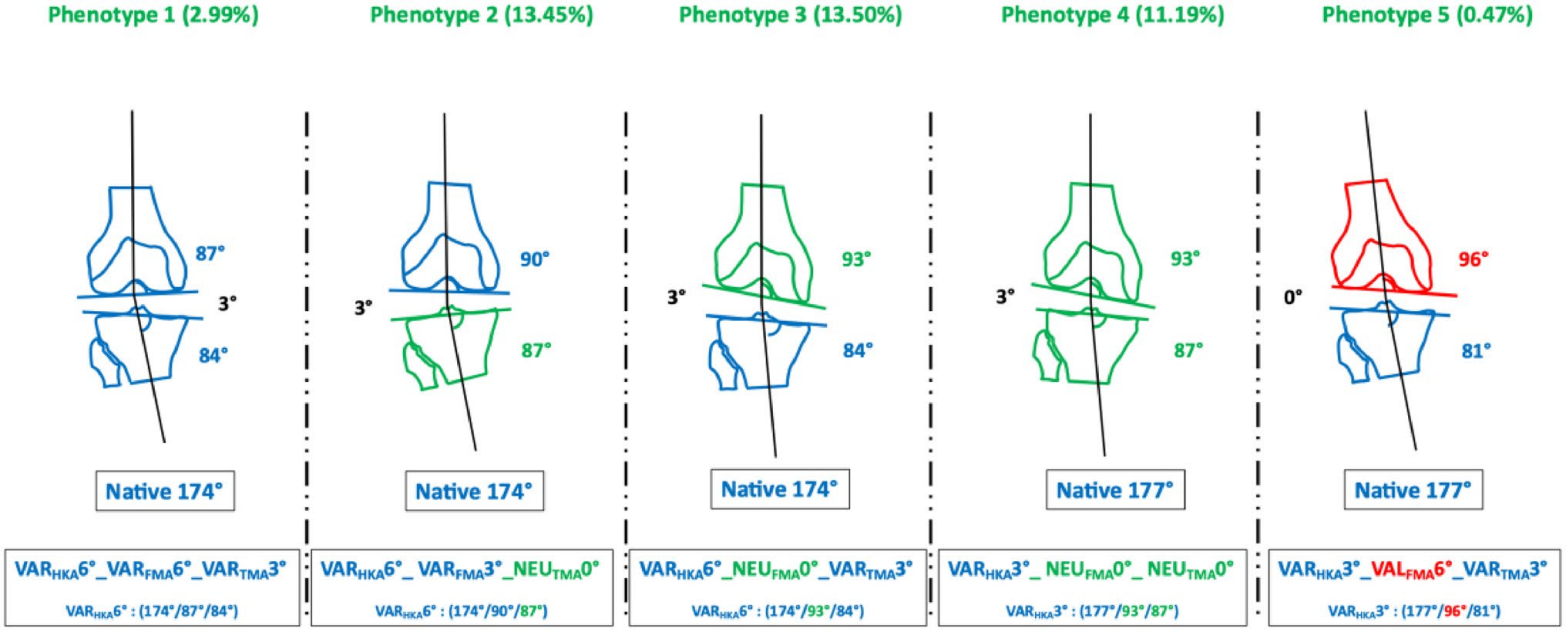
The five exemplary VAR “native” phenotypes

As categorising patients only according to the overall alignment of the leg, i.e. dividing them into varus, valgus and neutral patients, does not reflect the variability of coronal alignment, Hirschmann et al. introduced a system to categorise patients according to the alignment of the joint lines of the tibia (TMA) and femur (FMA) in relation to the overall alignment (HKA) [9]. The phenotypes are named in the following order. The first abbreviation (NEU, VAR, VAL) indicates the direction of alignment. The second (HKA, FMA and TMA) indicates the measured angle. This is followed third by the mean value of the alignment which covers a range of ± 1.5°. Figures 2 and 3 show five different phenotypes. The phenotypes were selected to represent the most common different possible combinations that can lead to a phenotype with varus limb alignment (FMA and TMA can be either NEU, VAL or VAR). All due care was taken to select the most frequent phenotypes from a cohort of 1904 patients with knee osteoarthritis and varus alignment (Table 1). The different simulations are performed for these specific knee phenotypes to better understand the trade-offs made after dogmatic realignment and their impact on gait pattern.

**Table 1:** Basic characteristics of the cohort of osteoarthritic patients

	Overall
Number of patients	1904
Age (years), mean (± SD)	70.6 (± 8.7)
Male gender, *n* (%)	868 (45.6%)

### TKA alignment strategies

Mechanical alignment (MA) aims to position both the femoral and tibial components perpendicular to the mechanical axis of the corresponding bone to achieve a HKA of 180°. A HKA deviation of ± 3° is considered acceptable (Table 2).

**Table 2:** Changes in the medial and lateral distal offset depending on the chosen alignment philosophy

		First VAR phenotype (VAR_HKA_6°)				
		HKA	FMA	TMA	Lateral condyle proximalisation	Medial tibia proximalisation
Preop alignment	Constitutional	174	87	84		
Postop alignment	Mechanical	180	90	90	3 mm	6 mm
	Anatomical		93	87	6 mm	3 mm
	Restricted	177	88	89	1 mm	5 mm
	Kinematic	174	87	84	0	0

The anatomical alignment (AA) technique has the goal to create an oblique joint line of 2–3° from the perpendicular to the mechanical axis, respectively, of 2–3° of valgus for the femur and 2–3° of varus for the tibia in relation to the mechanical axis [18]. The target value of the HKA is 180° (Table 3).

**Table 3:** Changes in the medial and lateral distal offset depending on the chosen alignment philosophy

		Second VAR phenotype (VAR_HKA_6°)				
		HKA	FMA	TMA	Lateral condyle proximalisation	Medial tibia proximalisation
Preop alignment	Constitutional	174	90	87		
Postop alignment	Mechanical	180	90	90	0	3 mm
	Anatomical		93	87	3 mm	0
	Restricted	180	90	87	0	0
	Kinematic					

The kinematic alignment (KA) technique aims to restore pre-arthritic limb and joint line alignment of TMA, FMA and HKA whilst sparing the ligamentous structures [12].

The restricted kinematic alignment (rKA) technique aims to restore constitutional joint lines and limb alignment, taking into account a safe zone, i.e. the HKA should remain ≤ 3° of 180° and the FMA and TMA should be ± 5° of 90° in relation to the mechanical axis [1].

According to the alignment strategy chosen, the different bone cuts may lead to a change in the joint line obliquity and to a change in the joint line height.

Thus, the joint line obliquity is defined as the angle formed by a parallel line to the floor and the joint line [10]. With regard to the change in joint line height, a distinction must be made between a symmetrical and an asymmetrical change in joint line height. There is evidence in the literature that a symmetrical change in joint line height can have negative effects on clinical outcome, but it is unclear what the consequences of an asymmetrical change are [27].

## Results

### Phenotype 1: VAR_HKA_174° VAR_FMA_87° VAR_TMA_84° (Fig. 4)

**Fig. 4:**
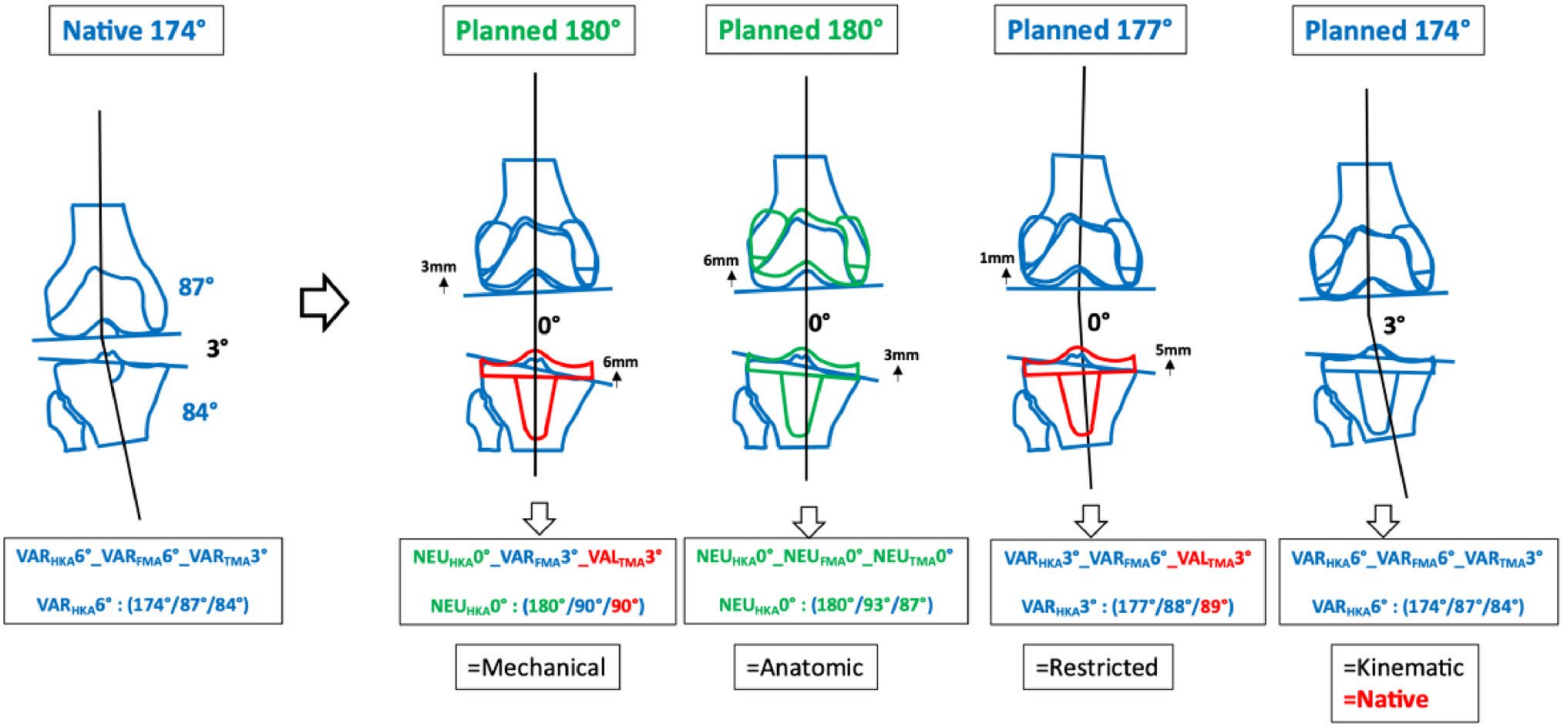
First main VAR “native” phenotype 1 VAR_HKA_174° VAR_FMA_87° VAR_TMA_84°

In the cohort described, the prevalence of this phenotype in the osteoarthritic varus population is 3% [6]. This phenotype has strongly oblique joint lines and, apart from KA, all alignment strategies change the alignment of the limb. The greatest alterations to the joint lines occur in MA and AA, where the medial tibial joint line is raised by 6 and 3 mm, respectively, and the lateral femoral condyle is shifted proximally by 3 or 6 mm, respectively.

### Phenotype 2: VAR_HKA_174° VAR_FMA_90° NEU_TMA_87° (Fig. 5)

**Fig. 5:**
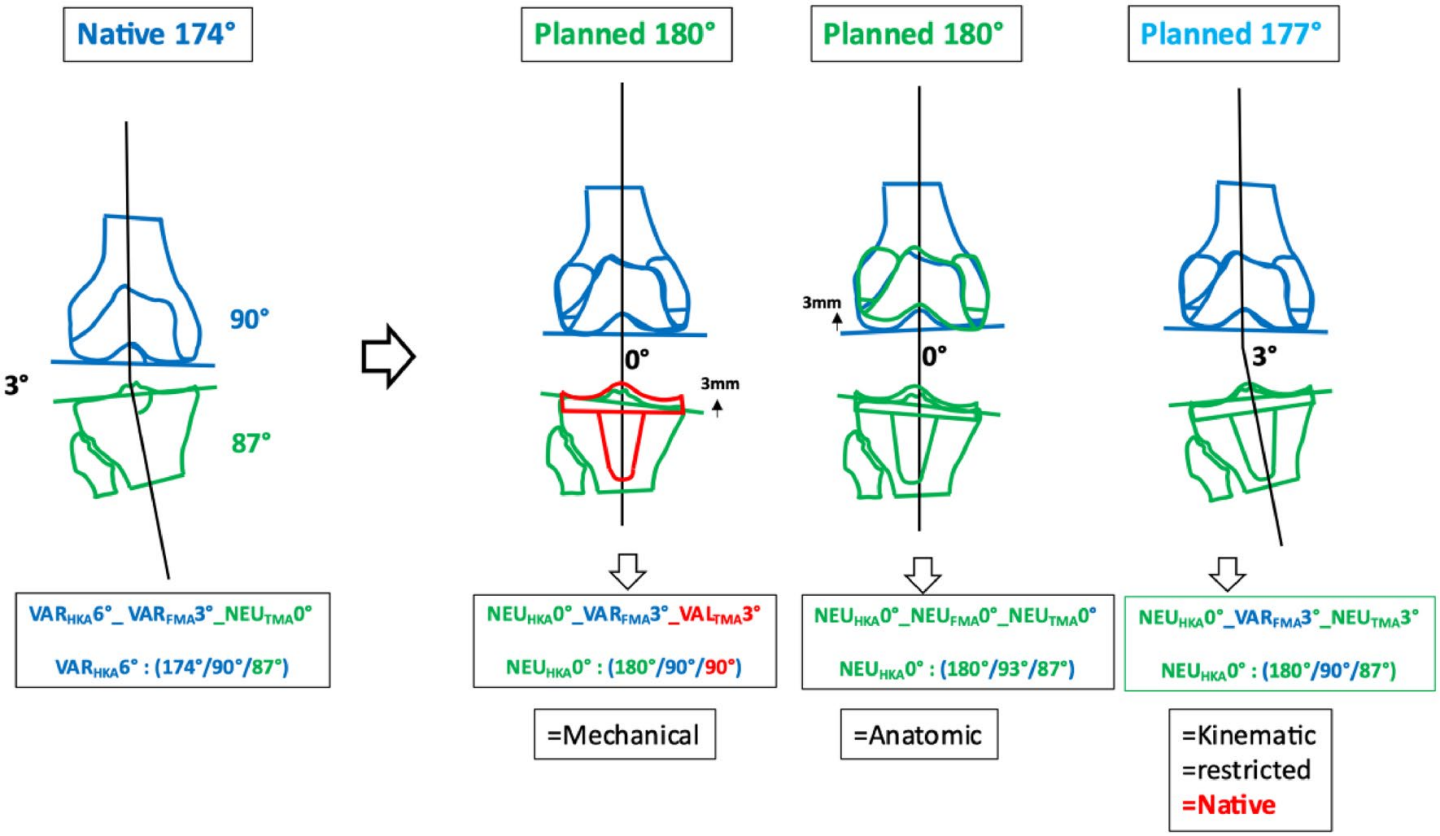
Second main NEU “native” phenotype VAR_HKA_174° VAR_FMA_90° NEU_TMA_87°

In the cohort described, the prevalence of this phenotype in the osteoarthritic varus population is 13.5% [6]. In this varus phenotype, only the femur is in a varus alignment. Changes to the joint lines are only required when MA and AA strategies are applied.

### Phenotype 3: VAR_HKA_174° NEU_FMA_93° VAR_TMA_84° (Fig. 6)

**Fig. 6:**
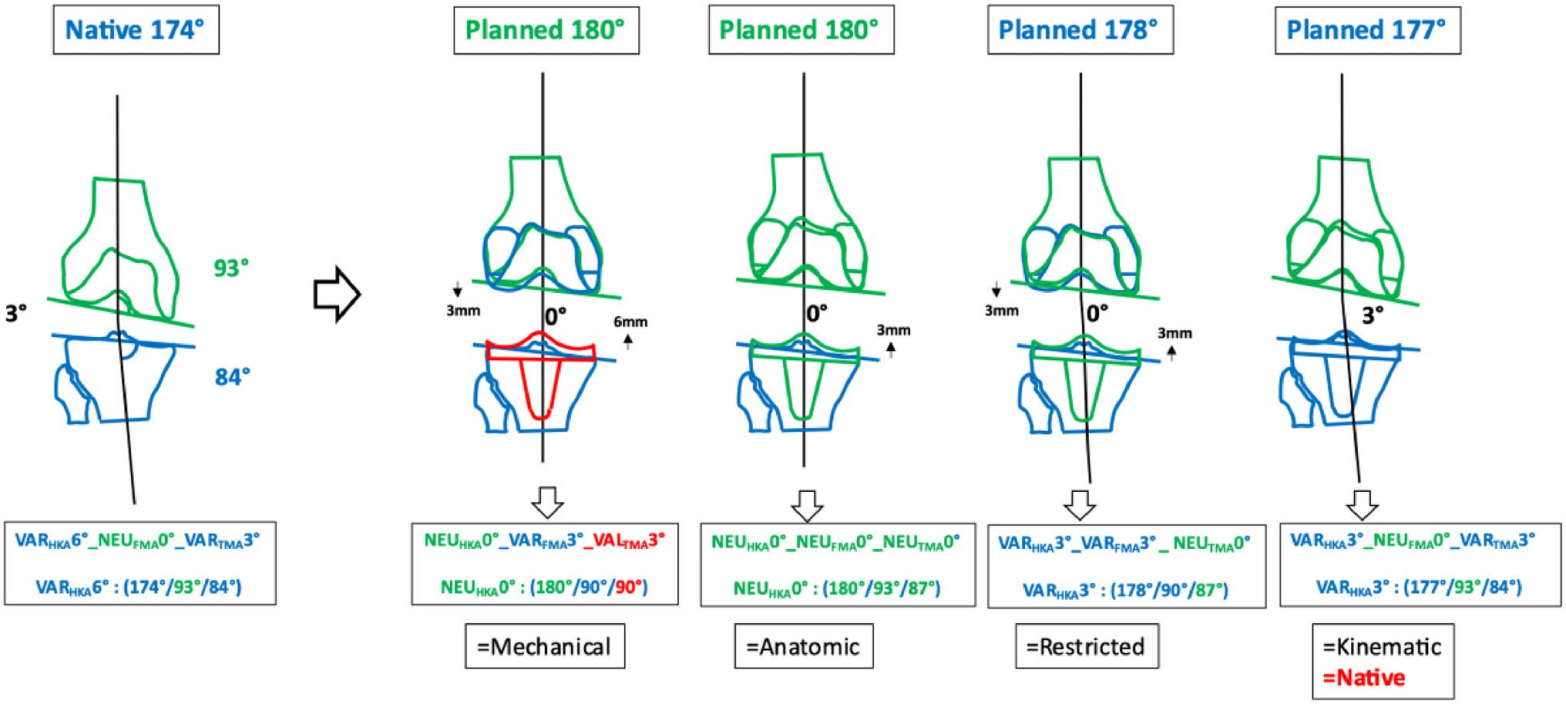
Third main NEU “native” VAR_HKA_174° NEU_FMA_93° VAR_TMA_84°

With a prevalence of 13.5% in the described varus population, this phenotype is rather frequent [6]. The largest bone cuts are seen when the MA is applied.

### Phenotype 4: VAR_HKA_177° NEU_FMA_93° NEU_TMA_87° (Fig. 7)

**Fig. 7:**
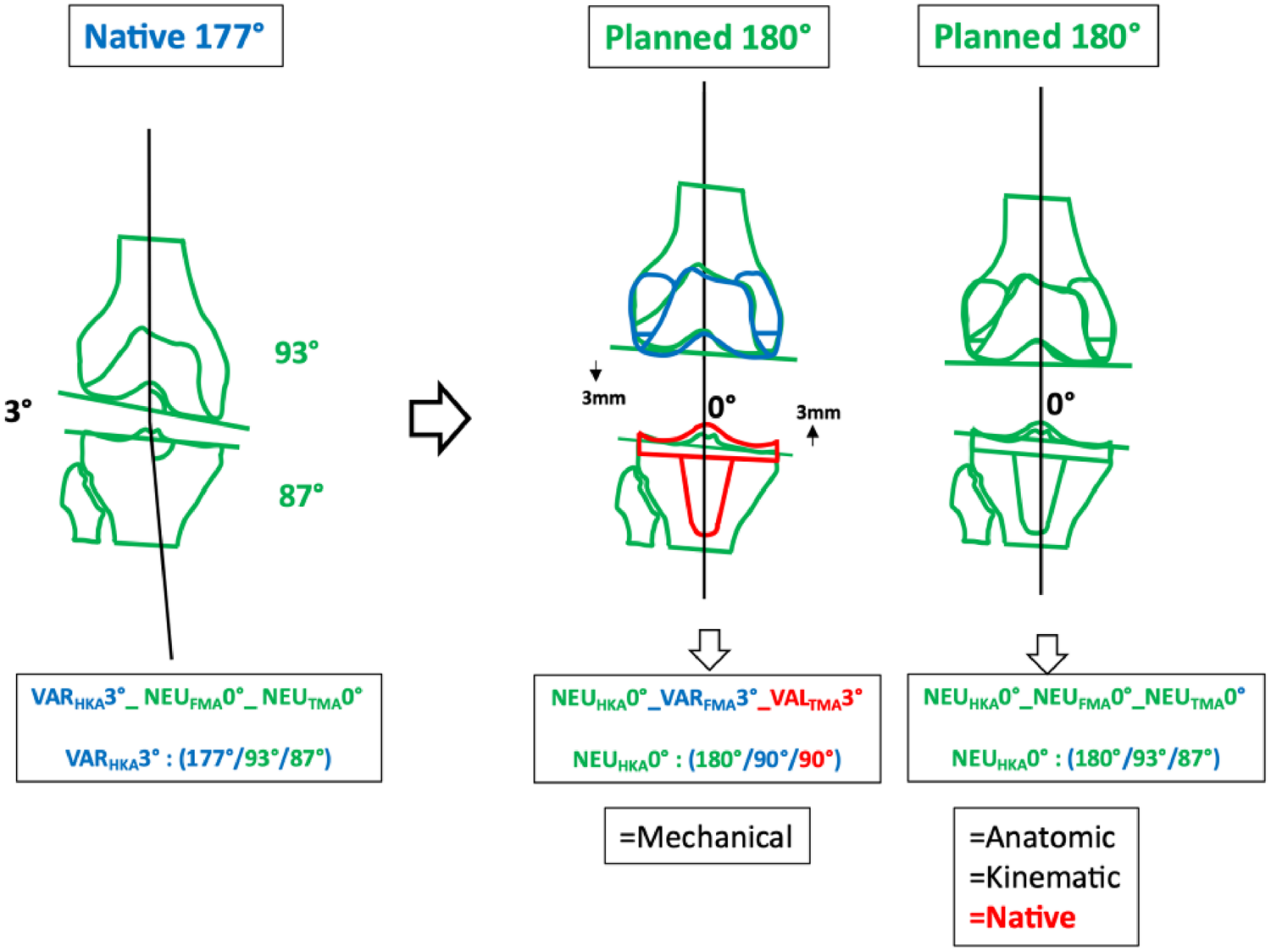
Fourth main NEU “native” phenotype VAR_HKA_177° NEU_FMA_93° NEU_TMA_87°

In the cohort described, the prevalence of this phenotype in the osteoarthritic varus population is 11.2% [6]. This phenotype corresponds to the alignment of the AA, and therefore only the MA requires changes to the joint line alignment.

### Phenotype 5: VAR_HKA_177° VAL_FMA_96° VAR_TMA_81° (Fig. 8)

**Fig. 8:**
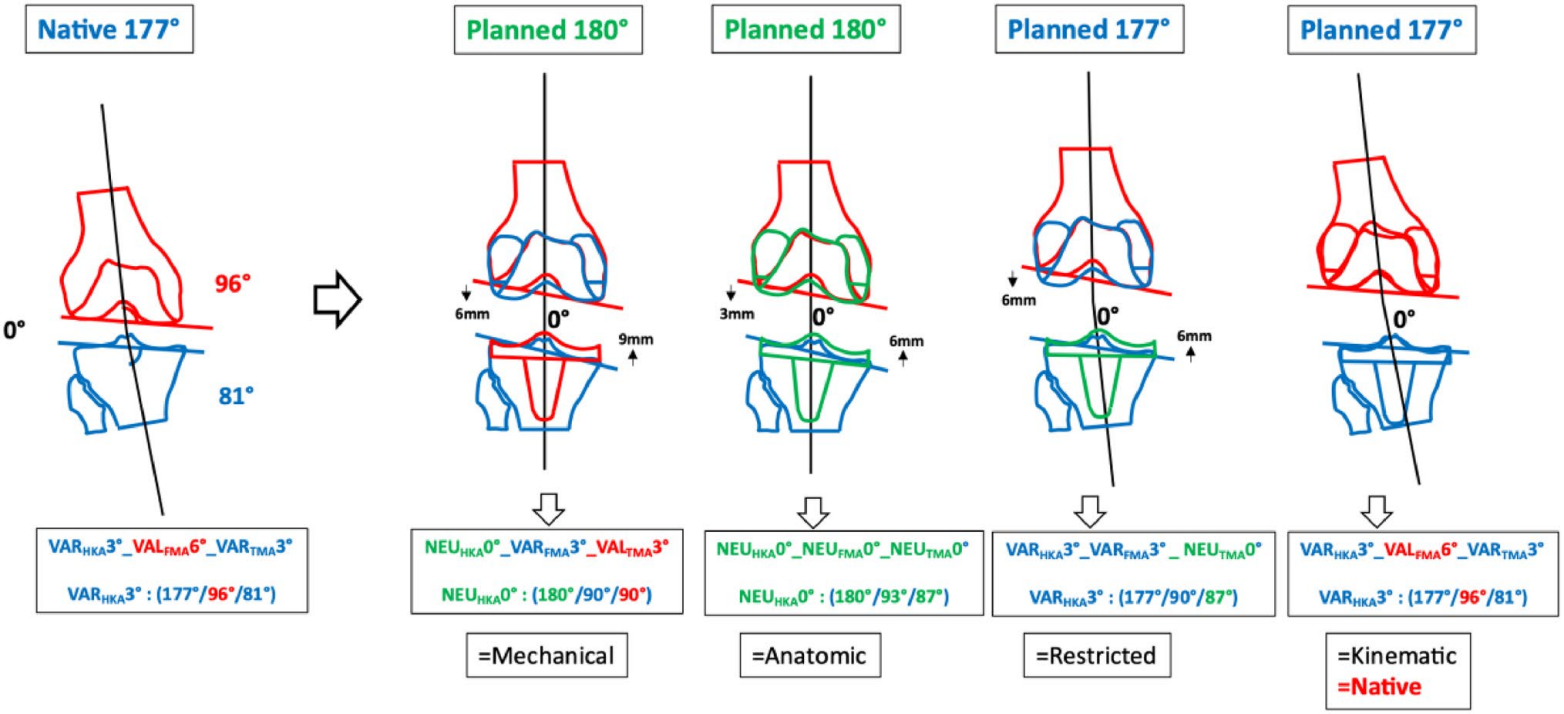
Fifth main VAR “native” phenotype VAR_HKA_177° VAL_FMA_96° VAR_TMA_81°

In the cohort described, the prevalence of this phenotype in the osteoarthritic varus population is 0.5% [6]. In this phenotype, despite the only moderate overall limb alignment of 3° varus, i.e. HKA 3°, all alignment strategies except KA require significant changes in joint lines due to the very oblique TMA and FMA.

## Discussion

The main findings of this study were that when only bone cuts are considered, the exemplary VAR phenotypes resulted in a variable change in the distal femoral joint line, offset and joint line obliquity depending on the alignment strategy chosen. It is well established that joint line changes occur frequently and should be avoided as much as possible [11]. The joint line changes currently reported are symmetrical and this is misleading or even more it can be called incorrect. As shown in the present study, the joint line changes are mostly asymmetrical, meaning that the change was not equal medially and laterally and often exceeded 3 mm in height. A distalisation of the lateral joint line might lead to overtensioning of the iliotibial tract in flexion and lateral and/or anterior compartment knee pain. When it comes to a stable prosthetic knee joint, it is possible to achieve it with sophisticated ligament balancing techniques. However, even when the knee joint is stable, the asymmetric changes of the joint line still lead to a distalised lateral femur and the associated problems remain. In fact, it results in altered loading of the patella as shown in a landmark study by Slevin et al. [25]. Furthermore, it also leads to altered kinematics in deep knee flexion [22]. One also needs to differentiate varus alignment in OA from constitutional varus alignment in a native knee as, in a constitutional varus, the joint line is parallel to the floor during gait whereas, in OA knees, it is not the case anymore [29] (Table 4).

**Table 4:** Changes in the medial and lateral distal offset depending on the chosen alignment philosophy

		Third VAR phenotype (VAR_HKA_6°)				
		HKA	FMA	TMA	Lateral condyle distalisation	Medial tibia proximalisation
Preop alignment	Constitutional	174	93	84		
Postop alignment	Mechanical	180	90	90	3 mm	6 mm
	Anatomical		93	87	0	3 mm
	Restricted	178	90	87	3 mm	3 mm
	Kinematic	177	93	84	0	0

If the knee would be mechanically aligned for phenotype 1 VAR_HKA_174° VAR_FMA_87° VAR_TMA_84°, the changes in component alignment from the native phenotype would be drastic, as the medial tibial plateau would be proximalised by 6 mm and the lateral femoral condyle would be shifted proximally by 3 mm. In consequence to achieve a balanced knee, this would require an extensive release medially, hence leading to an unpredictable change in laxity of the soft-tissue envelope. From Graichen et al., it is understood that the lateral extension gap (4.1 mm) was significantly larger than the medial extension gap (0.6 mm) in 657 (97%) patients with varus knees undergoing navigated TKA [4]. Moreover, women had significantly larger extension and flexion gaps [4]. The amount of varus deformity correlates highly with the medio-lateral gap difference in extension, but not in any flexion angle. Based on his findings, it is still unclear how much lateral laxity in extension a patient after TKA would tolerate. The discussion is ongoing. Sappey-Marinier et al. retrospectively investigated medial OA patients (*n* = 749 knees) who underwent KA TKA using standardised weight bearing long-leg and valgus stress radiographs. They found that using the KA philosophy, a well-balanced knee in extension can be achieved for varus knees [19]. A possible compromise for coronal alignment in the aforementioned case (VAR_HKA_174° VAR_FMA_87° VAR_TMA_84°) might offer rKA, which reduces the changes of preoperative alignment, but also the need for extensive ligament releases. Pure kinematic alignment does reconstruct the preoperative coronal alignment, but also currently pushes the surgeon over the border of what coronal alignment is allowed with most conventional TKA systems, which were mostly developed for mechanical alignment. A recent study has shown an increased risk of tibial loosening with restricted KA using conventional posterior-stabilised TKA [20]. This might be implant related and change when novel TKA systems are purely developed for kinematic alignment, but it raises concerns for a general unrestricted use of such alignment technique. Modifications regarding the trochlear opening angle, the anterior component thickness as well as the length of the anterior femoral shield should also be included in the discussion (Table 5).

**Table 5:** Changes in the medial and lateral distal offset depending on the chosen alignment philosophy

		Fourth VAR phenotype (VAR_HKA3_°)				
		HKA	FMA	TMA	Lateral condyle distalisation	Medial tibia proximalisation
Preop alignment	Constitutional	177	93	87		
Postop alignment	Mechanical	180	90	90	3 mm	3 mm
	Anatomical		93	87	0	0
	Restricted	180	93	87		
	Kinematic					

In phenotype 2 VAR_HKA_174° VAR_FMA_90° NEU_TMA_87°, the changes in joint line obliquity are only 3 mm regardless of the alignment strategy chosen.

In phenotype 3 VAR_HKA_174° NEU_FMA_93° VAR_TMA_84°, the optimal compromise is most likely between the alignment strategies rKA and AA, depending on what seems more important to the treating surgeon, namely an alignment that is as close to the native knee as possible or an overall bone alignment that is as neutral as possible.

Independently of the alignment strategy, phenotype 4 VAR_HKA_177° NEU_FMA_93° NEU_TMA_87° leads to only minor changes of the joint line. In this respect, the selection of the alignment strategy probably plays a less important role with regard to consequent clinical outcomes.

The phenotype 5 VAR_HKA_177° VAL_FMA_96° VAR_TMA_81° has only a slight varus total limb alignment. However, FMA and TMA are decisively oblique. Whilst this phenotype is rather rare, it is intended to demonstrate that it is in these rare joint configurations that there are major differences in the extent of resection between KA and the other alignment strategies. To avoid oblique implantation of the implants, significant resection changes and associated adjustments of the ligamentous apparatus would be required in rKA and more even more significantly in MA and AA. How much symmetrical and asymmetrical change in the height of the joint line is considered acceptable is not clear yet. However, van Lieshout et al. found frequent complications for symmetrical changes of more than 4 mm, and therefore recommend this as a relevant threshold [27]. To date, there are no widely accepted thresholds in the literature for the obliquity of component alignment in varus patients. However, in his 10-years follow-up study, Howell et al. [9] showed no negative correlation between component alignment and implant survival in unrestricted KA. Other long-term studies on the unrestricted use of KA are yet pending. Others have shown an increased tibial loosening rate in short-term for restricted KA, which raises concern about long-term survival rates [21]. This is in line with a more recent RSA study which has shown a correlation between tibial coronal alignment and increased base plate migration of the tibia. The findings were not seen for the whole limb alignment represented by HKA [26], which highlights the importance of assessing the detailed joint line configuration and not just the HKA. It, therefore, seems very important to categorise and study the knees preoperatively and postoperatively based on the individual component angles such as the phenotype concept used in the present study [8, 9]. In the absence of long-term studies examining the outcome of different alignment strategies for the different varus phenotypes, simulations such as the one presented here can assist the treating surgeon in determining the best alignment strategy for the individual patient. For the sake of the patient, a safe transition from mechanical alignment toward more personalised alignment is indicated [30]. A safe zone concept helps to safely extend coronal alignment positions from systematic to a more personalised alignment target [21] (Table 6).

**Table 6:** Changes in the medial and lateral distal offset depending on the chosen alignment philosophy

		Fifth VAR phenotype (VAR_HKA_3°)				
		HKA	FMA	TMA	Lateral condyle distalisation	Medial tibia proximalisation
Preop alignment	Constitutional	177	96	81		
Postop alignment	Mechanical	180	90	90	6 mm	9 mm
	Anatomical		93	87	3 mm	6 mm
	Restricted	178	90	87	6 mm	6 mm
	Kinematic	177	96	81	0	0

Nevertheless, this study has some limitations. Only the effects of specific alignment strategies on five exemplary varus phenotypes in the coronal plane were investigated. However, such simulations could be performed for more phenotypes in the future and be updated for the latest personalised alignment strategies as they are in constant development [3]. Changes in the alignment of the knee have an impact on the alignment of the ankle and the hip [5, 13, 17]. These changes, though, were not visualised as they were beyond the scope of this study. Moreover, the focus was laid on coronal alignment in a standing position with an extended leg, whereas sagittal and axial alignment of the implants were not investigated. For more clarity, the influence of alignment on flexion behaviour was not simulated.

## Conclusion

This simulation study shows that significantly different amounts of bone resection are required depending on the varus phenotype and the alignment strategy chosen. Based on the simulations performed, it can, therefore, be assumed that an individual decision for the respective phenotype is more important than the dogmatically correct alignment strategy. By including such simulations, the modern orthopaedic surgeon can now avoid biomechanically inferior alignments and still obtain the most natural possible knee alignment for the individual patient.

## Data Availability

Data is available at request in personal repository.
